# Risks of Major Mental Disorders and Irritable Bowel Syndrome among the Offspring of Parents with Irritable Bowel Syndrome: A Nationwide Study

**DOI:** 10.3390/ijerph18094679

**Published:** 2021-04-28

**Authors:** Ta-Chuan Yeh, Ya-Mei Bai, Shih-Jen Tsai, Tzeng-Ji Chen, Chih-Sung Liang, Mu-Hong Chen

**Affiliations:** 1 National Defense Medical Center, Department of Psychiatry, Tri-Service General Hospital, Taipei 114, Taiwan; fantine7520@ndmctsgh.edu.tw; 2 Department of Psychiatry, Penghu Branch, Tri-Service General Hospital, Penghu 880, Taiwan; 3 Institute of Brain Science, National Yang Ming Chiao Tung University, Taipei 112, Taiwan; 4 Department of Psychiatry, Taipei Veterans General Hospital, Taipei 112, Taiwan; ymbi@mail2000.com.tw (Y.-M.B.); sjtsai@VGHTPE.gov.tw (S.-J.T.); 5 Department of Psychiatry, College of Medicine, National Yang Ming Chiao Tung University, Taipei 112, Taiwan; 6 Institute of Hospital and Health Care Administration, National Yang Ming Chiao Tung University, Taipei 112, Taiwan; tjchen@vghtpe.gov.tw; 7 Department of Family Medicine, Taipei Veterans General Hospital, Taipei 112, Taiwan; 8 National Defense Medical Center, Department of Psychiatry, Beitou Branch, Tri-Service General Hospital, Taipei 112, Taiwan; 9 National Defense Medical Center, Graduate Institute of Medical Sciences, Taipei 114, Taiwan

**Keywords:** irritable bowel syndrome, neurodevelopmental disorders, mental disorders, genetic inheritance

## Abstract

Irritable bowel syndrome (IBS) is a functional bowel disorder that is highly comorbid with mental disorders. However, few studies have examined the risk of attention-deficit/hyperactivity disorder (ADHD), autism spectrum disorder (ASD), bipolar disorder, major depressive disorder (MDD), and schizophrenia in the offspring of parents with IBS. We used nationally representative cross-sectional survey data to analyze cross-generational transmission patterns of both IBS and major mental disorders. Odds ratio (OR) was calculated by using logistic regression models with adjustment for potential confounding factors. Offspring of parents with IBS were more likely to develop IBS themselves (OR = 2.41, 95% confidence interval (CI), 2.09–2.78), ADHD (OR = 1.33, 95% CI, 1.08–1.62), and MDD (OR = 1.32, 95% CI, 1.04–1.68) than the controls. Data stratification by parental sex revealed that paternal IBS increased risk of ADHD (OR = 1.34, 95% CI, 1.01–1.77) in the offspring, while maternal IBS increased the risk of MDD (OR = 1.51, 95% CI, 1.11–2.06). This is the first study to reveal parental IBS is associated with IBS, ADHD, and MDD among offspring, suggesting the necessity for early implementation of prevention strategies for at-risk children.

## 1. Introduction

Irritable bowel syndrome (IBS) is a common and chronic condition of the digestive system, with a prevalence rate ranging from of 2.4% to 31% [1]. The Rome IV criteria defines IBS as a condition of recurrent abdominal pain in conjunction with altered bowel habits including diarrhea, constipation, and mixed symptoms of constipation and diarrhea [2]. In clinical settings, IBS is the most common functional gastrointestinal disorder accounting for 25–50% of all referrals to gastroenterologists [3]. Because the spectrum and severity of the disorder can be debilitating to many patients, IBS leads to high medical costs and a significant impact on the quality of life [4,5].

Although the diagnostic criteria for IBS does not include psychological symptoms, somatization and depression often interplay with the disease course of IBS [6,7]. A growing body of literature has focused on the bidirectional relationship between IBS and mental disorders, and one of the most commonly implicated hypotheses is dysfunction of the gut-brain axis (GBA) which integrates stress responses, the gut microbiome, and immune regulation [8]. A cohort study with mother-child dyads revealed that repeated gastroenteritis during childhood was significantly associated with an increased prevalence of neurodevelopment disorders at 15.5 years of age, including attention-deficit/hyperactivity disorder (ADHD) [9]. Another study compared fecal microflora from autistic children and controls, and suggested that the increasing diversity of the microbiome in autistic children may be associated with their psychiatric- and IBS-related symptoms [10]. Another study reported that childhood IBS was an important medical comorbidity which contributed to the development of adult bipolar disorder [11].

In addition to a high tendency of coaggregation in twins [12] and probands [13], IBS might be an independent predictor of mental disorders within families [14]. An observational study suggested that IBS is independently associated with a familial history of mental disorders and may be linked to a history of alcohol or substance abuse [14]. Several genetic studies have indicated that the pathophysiology and treatment response of IBS are associated with monoamine transporter gene polymorphisms, such as those for serotonin [15] and norepinephrine [16], which contribute to the development of several mental disorders [17].

Although IBS can cause substantial distress and burden to the patients themselves, the link between parental IBS and the presence of mental disorders in their offspring, remains to be determined. A few studies have investigated the association between IBS and mental disorders through cross-generational transmission patterns. Previous study found repeated episodes of gastroenteritis were associated with emotional and behavioral difficulties in early life [9]. It also revealed that the episodes of gastroenteritis at childhood can significantly increase the risk for mental disorders in adolescents [9]. Another study showed maternal mental illness are strongly connected to the IBS in their children [14]. However, these studies used small sample sizes, had a high attrition rate [14], and the results were also limited by unvalidated IBS diagnoses [9] and not controlling for the presence of parental mental disorders [18].

In this study, we used data from the Taiwan National Health Insurance Research Database (NHIRD), which contains data from the entire Taiwanese population [19,20,21]. We examined the association between parental IBS and the risk of developing major mental disorders in their offspring. First, we investigate the co-occurrence of IBS and parental major mental disorders. Then, we further hypothesized that children born to parents with IBS were more likely to develop major mental disorders during the follow-up period, compared to children born to parents without IBS.

## 2. Materials and Methods

### 2.1. Data Source

The Taiwan NHIRD is audited and authorized by the National Health Research Institute for scientific and study purposes. The information of subjects included in the NHIRD are anonymized to maintain individual privacy. Comprehensive information on insured subjects in the NHIRD include their demographic data, dates of clinical visits, and disease diagnoses. Using a unique personal identification number assigned to each resident in Taiwan, all of the information is linked together.

The recorded family kinships listed in the NHIRD were validated and used for genealogical reconstruction [22,23]. The diagnostic codes used were based on the International Classification of Diseases, 9th Revision, Clinical Modification (ICD-9-CM). In Taiwan, several epidemiological studies assessing disease prevalence and population trends have used the information collected in the NHIRD [22,23,24,25]. The Taipei Veterans General Hospital Institutional Review Board approved this study (2018-07-016AC).

### 2.2. Inclusion Criteria of Parents with IBS and Their Offspring

Two types of parent–child cohorts (an IBS parent-child cohort and a non-IBS-parent-child cohort) were enrolled in our study to investigate the association between parental IBS and offspring risk of developing major mental disorders. Fathers or mothers, who were diagnosed with IBS (ICD-9-CM code: 564.1) by a board-certified gastroenterologists or colorectal specialists, at least twice, and had raised any biological child (son or daughter), were identified as an IBS parent-child cohort. A 1:4 case-control matched analysis was conducted based on age, sex, and familial relationship. For example, a 40-year-old mother with IBS who had an 8-year-old daughter was matched with four 40-year-old mothers without IBS who had an 8-year-old daughter.

### 2.3. Disease Classification and Assessment of Covariates

The major mental disorders identified were schizophrenia (ICD-9-CM code: 295), bipolar disorder (ICD-9-CM codes: 296, except for 296.2, 296.3, 296.9, and 296.82), major depressive disorder (MDD; ICD-9-CM codes: 296.2 and 296.3), autism spectrum disorder (ASD; ICD-9-CM code: 299), and ADHD (ICD-9-CM code: 314). These disorders were diagnosed at least twice by board-certified psychiatrists, based on their clinical judgment and diagnostic interviews. The assessed covariates included the mental disorders the parents were diagnosed with, the Charlson Comorbidity Index, the place of residence (classified into five categories according to the level of urbanization), and monthly income.

### 2.4. Statistical Methods

Chi-square statistics and F-tests were used to compare categorical and continuous variables, respectively, between IBS-parent-child and non-IBS-parent-child cohorts. Two logistic regression models were performed to correlate the occurrence of parental IBS and the offspring risk of developing major mental disorders. Model 1 was adjusted for the demographic data, including the ages of parents and children, sex of the parents and children, residence level of urbanization, and income. Model 2 was further adjusted for the incidence of parental mental disorders, including schizophrenia, bipolar disorder, and MDD. Due to the rare incidence, we did not adjust for parental ADHD (*n* = 14, 0.03%) or ASD (*n* = 2, 0.005%) in the regression model. Sub-analyses stratified by paternal or maternal data were also performed. In addition, a logistic regression model adjusted for demographic data and the presence of parental mental disorders was further used to examine how parental IBS influenced the risk of offspring having IBS. A 2-tailed *p*-value of less than 0.05 was considered statistically significant. All data processing and statistical analyses were performed with Statistical Package for Social Science (SPSS) version 17 software (SPSS Inc., IBM, Armonk, NY, USA) and Statistical Analysis Software (SAS) version 9.1 (SAS Institute, Cary, NC, USA).

## 3. Results

The parents with IBS were more likely to have mental disorders than the non-IBS-parents in the control cohort, which included schizophrenia (0.6% vs. 0.4%, *p* = 0.004), bipolar disorder (0.9% vs. 0.4%, *p* < 0.001), and MDD (4.3% vs. 1.4%, *p* < 0.001). The children born to IBS parents were more likely to have ADHD (1.6% vs. 1.2%, *p* = 0.003) and MDD (1.1% vs. 0.8%, *p* = 0.006) than the children born to parents without IBS. We included 8377 IBS parent-child cohorts and 33,508 age-, sex-, and kinship-matched non-IBS-parent-child cohorts. The other demographic data, including age, sex, place of residence, and income status are listed in Table 1 and were adjusted for in model 1 and model 2.

We next examined the risk of developing major mental disorders associated with parental history of IBS (Table 2). In model 1, with adjustment for the demographic data, parental IBS was associated with an increased risk of developing ADHD (odds ratio (OR) = 1.37, 95% confidence interval (CI) = 1.12–1.67) and MDD (OR = 1.40, 95% CI = 1.11–1.77), in the children. The associations remained statistically significant in model 2, which was further adjusted for the presence of parental mental disorders. The ORs were 1.33 (95% C I = 1.08–1.62) for ADHD and 1.32 (95% CI = 1.04–1.68) for MDD. We did not find any association between parental IBS with other mental disorders, including ASD, bipolar disorder, and schizophrenia. For IBS, parental history of IBS was associated with a two-fold higher risk in their offspring after adjusting for demographic data and the occurrence of parental mental disorders (OR = 2.41 in model 1; OR = 2.38 in model 2).

Lastly, we examined the effect of parental sex which might differentially contribute to disease risk (Table 3). After stratification by parental sex, children born to fathers with IBS were more likely to develop ADHD compared with controls (OR = 1.34, 95% CI = 1.01–1.77), while this risk was not present in children born to mothers with IBS compared with controls (OR = 1.31, 95% CI = 0.98–1.76). On the contrary, children born to mothers with IBS were more likely to develop MDD compared with controls (OR = 1.51, 95% CI = 1.11–2.06), while children born to fathers with IBS were not at higher risk compared with controls (OR = 1.09, 95% CI = 0.74–1.60). We did not observe an effect of parental sex on the risk of developing IBS. Both paternal and maternal IBS were significantly associated with an approximately two-fold higher risks of IBS occurring in their offspring compared with controls (OR = 2.15 for paternal IBS and 2.57 for maternal IBS).

## 4. Discussion

Previous studies have found a bidirectional relationship between IBS and mental disorders [9,14,18], while our study is the first one to examine this relationship in the context of a cross-generational transmission pattern. We compared IBS-parent-child cohorts with non-IBS-parent-child control cohorts and found that parental IBS was significantly associated with higher risks in the offspring for developing IBS and major mental disorders. After adjusting for the occurrence of parental major mental disorders and other demographic data, the risk was increased for ADHD, MDD, and IBS in the offspring of parents with IBS versus in those of parents without IBS. Additionally, the cross-generational transmission pattern was different for fathers and mothers with IBS. We found that paternal IBS was associated with a risk of having ADHD, while maternal IBS was associated with the risk of having MDD. However, both fathers and mothers with IBS seemed to contribute similarly to the risk of developing IBS in the offspring. In summary, we suggest that IBS is an independent risk factor for the development of both IBS and major mental disorders in the offspring.

Consistent with the literature, our findings support the hypothesis that parental history of IBS is associated with both gastrointestinal symptoms and psychological symptoms in their offspring [26,27]. A previous study conducted on twins, indicated that having parents with IBS is a stronger predictor than having a twin with IBS, implying that parental IBS could be a significant environmental factor precipitating the development of IBS [12]. This study also implicated parental history of IBS as a vulnerability factor for the development of stress-related disorders. Importantly, patients with IBS who experience early life stress, tend to have higher cortisol levels than healthy controls after a visceral stressor [28]. Among patients with IBS, those having a faster stress response and a slower recovery of stimulated cortisol displayed more IBS symptoms and overall poor quality of life [28]. In animal research, perinatal stress exposure facilitated the development of stress-induced visceral hypersensitivity, increased HPA response, and anxiety behavior in adult rats [29,30]. There is also a growing body of evidence suggesting a similar pattern of heart rate variability, sympathetic activation, and parasympathetic inhibition for patients with IBS, MDD, and ADHD [31,32,33,34,35]. These findings support the hypothesis that parental IBS plays an important role in developing major mental disorders and mediating life-long stress responses through the GBA. Therefore, children with a history of parental IBS may face either IBS-related physical discomfort or psychological distress from their parents, starting at an early age. These daily challenges might elicit HPA hyperactivity and autonomic dysregulation which are associated with the pathophysiology of these major mental disorders [31,32,36,37].

Previous family [13,14] and twin studies [12,18,38] have reported familial aggregation of IBS. Our study observed co-aggregation of IBS and major mental disorders in patients with a history of parental IBS; we suggest that a genetic predisposition may explain such relationships. Serotonin plays a critical role in controlling gastrointestinal motility and evidence suggests that disturbances in serotonin synthesis and metabolism contribute to the pathophysiology of bowel function in IBS [39]. A variety of psychiatric symptoms have been linked to a reduction of serotonin neurotransmission, including inattention, hyperactivity, and depression. Importantly, genetic studies have indicated a common single-nucleotide polymorphism in the promoter for synthesis of the serotonin transporter in depressive disorder and the diarrhea subtype of IBS [39,40]. Studies in patients with IBS also revealed an association between quality of life/psychological distress and polymorphisms of tryptophan hydroxylase (TPH), a rate-limiting enzyme in the biosynthesis of serotonin [41]. A nucleotide polymorphism of TPH has also been implicated in ADHD [42] and MDD [43]. Notably, a study by Sheehan et al. found an association between TPH polymorphism and ADHD, and this association was enhanced after data stratification by parental sex (OR = 3.7) [42], which is consistent with our finding of prominent paternal transmission in ADHD. In brief, the aforementioned findings support the hypothesis that family co-aggregation of IBS and MDD may be associated with several shared genetic factors.

A previous study using small sample sizes has suggested that there exists a relationship between IBS and mental disorders [9]. Using a nationwide population-based study design, this study found that parental IBS was a significant predictor of the diagnoses of ADHD and MDD in their offspring. Interestingly, a co-twin control study examined the genetic association and co-occurrence of MDD and IBS [38]. The findings from this study suggest that there is a higher risk for developing MDD, among IBS discordant monozygotic twins compared to control twins (OR = 3.17), implying that there is a common causal pathway between IBS and MDD [38]. Another study described a trend in depressive disorder (OR = 1.9) among twins with IBS compared to those without IBS [18]. These studies also suggested an interplay between familial and environmental factors in the pathophysiology mediating the co-occurrence of MDD and IBS [18]. A longitudinal cohort study examined the risk of behavioral difficulties after exposure to gastroenteritis in early childhood (between 30 and 36 months of age), and the authors also found that repeated gastroenteritis was significantly associated with hyperactivity/inattention symptoms and a later diagnosis of ADHD in adolescence (OR = 1.08) [9].

The generalizability of our results is subject to certain limitations. First, the prevalence rate of IBS in the current study was significantly lower than the rates found in Western countries [1]. An underestimation may be considered because only patients with IBS who suffered from severe symptoms might seek medical consultation and be included in the database. However, the prevalence of IBS might be higher when symptoms are obtained by questionnaires and patient interviews [1]. The diagnosis of IBS in our study was confirmed by board-certified gastroenterologists or colorectal specialists, at least twice, potentially yielding a more reliable diagnosis than a questionnaire-based diagnosis. Second, the current study did not evaluate the sub-type of IBS. Since a specific diagnostic code for diarrhea- or constipation- predominant IBS is not available in the ICD-9-code system, we were unable to examine these subtypes. Third, our study only focused on the influence of parental IBS on ADHD, ASD, bipolar disorder, schizophrenia, and MDD. Whether parental IBS may be a risk factor for other psychiatric disorders, such as anxiety spectrum disorders, requires further investigation. Fourth, though our results suggest that parental history of IBS is an important factor in offspring risk of major mental disorders, the causal association between these disorders remains to be determined. Fifth, the heritability of paternally transmitted vs. maternally transmitted risks of IBD and mental disorders might have been confounded by non-paternity. However, the data of non-paternity in Taiwan is not available. According to the previous studies, the estimate of non-paternity rate in Switzerland and German are approximately 0.64% and 1%, respectively [44]. Therefore, the confounding effect of non-paternity might be relatively small. Additionally, the bidirectional relationship between the digestive system and the brain were not directly examined in the current study.

## 5. Conclusions

In conclusion, children and adolescents with a history of parental IBS may be at a higher risk of developing IBS, ADHD, and MDD, and such associations seem to differ in relation to fathers and mothers. Previous studies have suggested the co-aggregation of IBS within families [13,14], our findings further support the prevalence of IBS and major mental disorders through both genetic and familial environment influences. Our findings not only indicate an increased risk of IBS, ADHD, and MDD in the offspring of parents with IBS but also that paternal IBS increased the risk of ADHD while maternal IBS increased the risk of MDD.

Furthermore, we found that this association remained significant after adjusting parental mental disorders, which suggests early recognition of psychosocial stressors in offspring of parents with IBS may be an essential prevention strategy for lowering their risk of IBS and mental disorders. Further research should be carried out to delineate the biopsychosocial model which triggers IBS and mental disorders among this particularly vulnerable population. This is the first study to reveal parental IBS is associated with a higher risk of IBS, ADHD, and MDD among offspring, suggesting the necessity for early implementation of prevention strategies for at-risk children.

## Figures and Tables

**Table 1:** Demographic data and prevalence of mental disorders among the offspring of parents with or without IBS.

Demographic Characteristics	Parents with IBS and Their Children (*n* = 8377)	Parents without IBS and Their Children (*n* = 33,508)	*p*
Age of parents (years, SD)	56.92 (11.62)	56.91 (11.63)	0.931
Sex of parents (*n*, %)			1.000
Male (father)	4108 (49.0)	16,432 (18.0)	
Female (mother)	4269 (51.0)	17,076 (51.0)	
Age of children (years, SD)	28.58 (11.92)	28.59 (11.91)	0.942
Sex of children (*n*, %)			0.883
Male (son)	4560 (54.4)	18,208 (54.3)	
Female (daughter)	3817 (45.6)	15,300 (45.7)	
Mental disorders of parents (*n*, %)			
Schizophrenia	52 (0.6)	129 (0.4)	**0.004**
Bipolar disorder	72 (0.9)	123 (0.4)	**<0.001**
Major depressive disorder	360 (4.3)	468 (1.4)	**<0.001**
IBS of children (*n*, %)	312 (3.7)	536 (1.6)	**<0.001**
Mental disorders of children (*n*, %)			
ADHD	134 (1.6)	397 (1.2)	**0.003**
ASD	19 (0.2)	62 (0.2)	0.412
Schizophrenia	54 (0.6)	207 (0.6)	0.762
Bipolar disorder	32 (0.4)	105 (0.3)	0.337
Major depressive disorder	94 (1.1)	270 (0.8)	**0.006**
Level of urbanization (*n*, %)			1.000
1 (most urbanized)	1876 (22.4)	7504 (22.4)	
2	3012 (36.0)	12,048 (36.0)	
3	1022 (12.2)	4088 (12.2)	
4	826 (9.9)	3304 (9.9)	
5 (most rural)	1641 (19.6)	6564 (19.6)	
Income-related insured amount			1.000
≤15,840 NTD/month	3288 (39.3)	13,152 (39.3)	
15,841~25,000 NTD/month	2381 (28.4)	9524 (28.4)	
≥25,001 NTD/month	2708 (32.3)	10,832 (32.3)	

ADHD attention deficit hyperactivity disorder; ASD autism spectrum disorder; IBS irritable bowel syn-drome; SD standard deviation; **Bold** type indicates the statistical significance.

**Table 2:** Logistic regression model of parental IBS and mental disorders of their children.

Variables	Mental Disorders of Children (OR, 95% CI)					IBS of Children (OR, 95% CI)
	ADHD	ASD	Schizophrenia	Bipolar Disorder	MDD	
Model 1						
Parental IBS						
Presence	**1.37 (1.12–1.67)**	1.22 (0.73–2.04)	1.04 (0.77–1.41)	1.22 (0.82–1.82)	**1.40 (1.11–1.77)**	**2.41 (2.09–2.78)**
Absence	1 (ref.)	1 (ref.)	1 (ref.)	1 (ref.)	1 (ref.)	1 (ref.)
Model 2						
Parental IBS						
Presence	**1.33 (1.08–1.62)**	1.20 (0.71–2.01)	1.02 (0.75–1.38)	1.10 (0.74–1.65)	**1.32 (1.04–1.68)**	**2.38 (2.06–2.75)**
Absence	1 (ref.)	1 (ref.)	1 (ref.)	1 (ref.)	1 (ref.)	1 (ref.)

Model 1: adjusted for demographic data. Model 2: adjusted for demographic data and parental mental disorders. ADHD attention deficit hyperactivity disorder; ASD autism spectrum disorder; CI confidence interval; IBS irritable bowel syndrome; MDD major depressive disorder; OR odds ratio; SD standard deviation. **Bold** type indicates the statistical significance.

**Table 3:** Logistic regression model of parental IBS and mental disorders of their children, stratified by parental sex*.

Variables	Mental Disorders of Children (OR, 95% CI)					IBS of Children (OR, 95% CI)
	ADHD	ASD	Schizophrenia	Bipolar Disorder	MDD	
Paternal IBS						
Presence	**1.34 (1.01–1.77)**	0.89 (0.41–1.92)	0.82 (0.50–1.36)	1.07 (0.58–1.92)	1.09 (0.74–1.60)	**2.15 (1.72–2.69)**
Absence	1 (ref.)	1 (ref.)	1 (ref.)	1 (ref.)	1 (ref.)	1 (ref.)
Maternal IBS						
Presence	1.31 (0.98–1.76)	1.59 (0.78–3.26)	1.18 (0.80–1.73)	1.11 (0.65–1.91)	**1.51 (1.11–2.06)**	**2.57 (2.13–3.11)**
Absence	1 (ref.)	1 (ref.)	1 (ref.)	1 (ref.)	1 (ref.)	1 (ref.)

Model 1: adjusted for demographic data. Model 2: adjusted for demographic data and parental mental disorders. ADHD attention deficit hyperactivity disorder; ASD autism spectrum disorder; CI confidence interval; IBS irritable bowel syndrome; MDD major depressive disorder; OR odds ratio; SD standard deviation. **Bold** type indicates the statistical significance.

## Data Availability

The NHIRD was released and audited by the Department of Health and Bureau of the NHI Program for scientific research (https://nhird.nhri.org.tw/, accessed on 27 April 2021). NHIRD can be obtained through the filing of a formal application that is regulated by the Department of Health and Bureau of the NHI Program.

## References

[1] Lovell R.M., Ford A.C. (2012). Global prevalence of and risk factors for irritable bowel syndrome: A meta-analysis. Clin. Gastroenterol. Hepatol..

[2] Drossman D.A. (2016). Functional Gastrointestinal Disorders: History, Pathophysiology, Clinical Features and Rome IV. Gastroenterology.

[3] Everhart J.E., Renault P.F. (1991). Irritable bowel syndrome in office-based practice in the United States. Gastroenterology.

[4] Enck P., Aziz Q., Barbara G., Farmer A.D., Fukudo S., Mayer E.A., Niesler B., Quigley E.M., Rajilic-Stojanovic M., Schemann M. (2016). Irritable bowel syndrome. Nat. Rev. Dis. Primers.

[5] Sandler R.S., Everhart J.E., Donowitz M., Adams E., Cronin K., Goodman C., Gemmen E., Shah S., Avdic A., Rubin R. (2002). The burden of selected digestive diseases in the United States. Gastroenterology.

[6] Lee C., Doo E., Choi J.M., Jang S.H., Ryu H.S., Lee J.Y., Oh J.H., Park J.H., Kim Y.S., Brain-Gut Axis Research Group of Korean Society of Neurogastroenterology and Motility (2017). The Increased Level of Depression and Anxiety in Irritable Bowel Syndrome Patients Compared with Healthy Controls: Systematic Review and Meta-analysis. J. Neurogastroenterol. Motil..

[7] Hollier J.M., van Tilburg M.A.L., Liu Y., Czyzewski D.I., Self M.M., Weidler E.M., Heitkemper M., Shulman R.J. (2019). Multiple psychological factors predict abdominal pain severity in children with irritable bowel syndrome. Neurogastroenterol. Motil..

[8] Bonaz B.L., Bernstein C.N. (2013). Brain-gut interactions in inflammatory bowel disease. Gastroenterology.

[9] Parent C., Pokhvisneva I., Gaudreau H., Meaney M.J., Silveira P.P. (2019). Association between Repeated Episodes of Gastroenteritis and Mental Health Problems in Childhood and Adolescence. J. Am. Acad. Child. Adolesc. Psychiatry.

[10] Finegold S.M., Dowd S.E., Gontcharova V., Liu C., Henley K.E., Wolcott R.D., Youn E., Summanen P.H., Granpeesheh D., Dixon D. (2010). Pyrosequencing study of fecal microflora of autistic and control children. Anaerobe.

[11] Post R.M., Altshuler L.L., Leverich G.S., Frye M.A., Suppes T., McElroy S.L., Keck P.E., Nolen W.A., Kupka R.W., Grunze H. (2013). Role of childhood adversity in the development of medical co-morbidities associated with bipolar disorder. J. Affect. Disord..

[12] Levy R.L., Jones K.R., Whitehead W.E., Feld S.I., Talley N.J., Corey L.A. (2001). Irritable bowel syndrome in twins: Heredity and social learning both contribute to etiology. Gastroenterology.

[13] Saito Y.A., Petersen G.M., Larson J.J., Atkinson E.J., Fridley B.L., de Andrade M., Locke G.R., Zimmerman J.M., Almazar-Elder A.E., Talley N.J. (2010). Familial aggregation of irritable bowel syndrome: A family case-control study. Am. J. Gastroenterol..

[14] Knight J.R., Locke G.R., Zinsmeister A.R., Schleck C.D., Talley N.J. (2015). Family history of mental illness or alcohol abuse and the irritable bowel syndrome. J. Psychosom. Res..

[15] Li Y., Nie Y., Xie J., Tang W., Liang P., Sha W., Yang H., Zhou Y. (2007). The association of serotonin transporter genetic polymorphisms and irritable bowel syndrome and its influence on tegaserod treatment in Chinese patients. Dig. Dis. Sci..

[16] Kim H.J., Camilleri M., Carlson P.J., Cremonini F., Ferber I., Stephens D., McKinzie S., Zinsmeister A.R., Urrutia R. (2004). Association of distinct alpha(2) adrenoceptor and serotonin transporter polymorphisms with constipation and somatic symptoms in functional gastrointestinal disorders. Gut.

[17] Hahn M.K., Blakely R.D. (2002). Monoamine transporter gene structure and polymorphisms in relation to psychiatric and other complex disorders. Pharmacogenom. J..

[18] Bengtson M.B., Aamodt G., Vatn M.H., Harris J.R. (2015). Co-occurrence of IBS and symptoms of anxiety or depression, among Norwegian twins, is influenced by both heredity and intrauterine growth. BMC Gastroenterol..

[19] Huang J.S., Yang F.C., Chien W.C., Yeh T.C., Chung C.H., Tsai C.K., Tsai S.J., Yang S.S., Tzeng N.S., Chen M.H. (2021). Risk of Substance Use Disorder and Its Associations With Comorbidities and Psychotropic Agents in Patients With Autism. JAMA Pediatr..

[20] Liang C.S., Bai Y.M., Hsu J.W., Huang K.L., Ko N.Y., Chu H.T., Yeh T.C., Tsai S.J., Chen T.J., Chen M.H. (2020). The Risk of Sexually Transmitted Infections Following First-Episode Schizophrenia Among Adolescents and Young Adults: A Cohort Study of 220 545 Subjects. Schizophr. Bull..

[21] Liang C.S., Chung C.H., Ho P.S., Tsai C.K., Chien W.C. (2018). Superior anti-suicidal effects of electroconvulsive therapy in unipolar disorder and bipolar depression. Bipolar Disord..

[22] Chen M.H., Hsu J.W., Huang K.L., Su T.P., Li C.T., Lin W.C., Tsai S.J., Cheng C.M., Chang W.H., Pan T.L. (2019). Risk and coaggregation of major psychiatric disorders among first-degree relatives of patients with bipolar disorder: A nationwide population-based study. Psychol. Med..

[23] Cheng C.M., Chang W.H., Chen M.H., Tsai C.F., Su T.P., Li C.T., Tsai S.J., Hsu J.W., Huang K.L., Lin W.C. (2018). Co-aggregation of major psychiatric disorders in individuals with first-degree relatives with schizophrenia: A nationwide population-based study. Mol. Psychiatry.

[24] Chen M.H., Lan W.H., Hsu J.W., Huang K.L., Su T.P., Li C.T., Lin W.C., Tsai C.F., Tsai S.J., Lee Y.C. (2016). Risk of Developing Type 2 Diabetes in Adolescents and Young Adults With Autism Spectrum Disorder: A Nationwide Longitudinal Study. Diabetes Care.

[25] Chen M.H., Hsu J.W., Huang K.L., Bai Y.M., Ko N.Y., Su T.P., Li C.T., Lin W.C., Tsai S.J., Pan T.L. (2018). Sexually Transmitted Infection Among Adolescents and Young Adults With Attention-Deficit/Hyperactivity Disorder: A Nationwide Longitudinal Study. J. Am. Acad. Child Adolesc. Psychiatry.

[26] Kanazawa M., Endo Y., Whitehead W.E., Kano M., Hongo M., Fukudo S. (2004). Patients and nonconsulters with irritable bowel syndrome reporting a parental history of bowel problems have more impaired psychological distress. Dig. Dis. Sci..

[27] Gunnar M.R., Cheatham C.L. (2003). Brain and behavior interface: Stress and the developing brain. Infant. Ment. Health J..

[28] Videlock E.J., Adeyemo M., Licudine A., Hirano M., Ohning G., Mayer M., Mayer E.A., Chang L. (2009). Childhood trauma is associated with hypothalamic-pituitary-adrenal axis responsiveness in irritable bowel syndrome. Gastroenterology.

[29] Coutinho S.V., Plotsky P.M., Sablad M., Miller J.C., Zhou H., Bayati A.I., McRoberts J.A., Mayer E.A. (2002). Neonatal maternal separation alters stress-induced responses to viscerosomatic nociceptive stimuli in rat. Am. J. Physiol. Gastrointest. Liver Physiol..

[30] Gareau M.G., Jury J., Yang P.C., MacQueen G., Perdue M.H. (2006). Neonatal maternal separation causes colonic dysfunction in rat pups including impaired host resistance. Pediatr. Res..

[31] Robe A., Dobrean A., Cristea I.A., Pasarelu C.R., Predescu E. (2019). Attention-deficit/hyperactivity disorder and task-related heart rate variability: A systematic review and meta-analysis. Neurosci. Biobehav. Rev..

[32] Griffiths K.R., Quintana D.S., Hermens D.F., Spooner C., Tsang T.W., Clarke S., Kohn M.R. (2017). Sustained attention and heart rate variability in children and adolescents with ADHD. Biol. Psychol..

[33] Cain K.C., Jarrett M.E., Burr R.L., Hertig V.L., Heitkemper M.M. (2007). Heart rate variability is related to pain severity and predominant bowel pattern in women with irritable bowel syndrome. Neurogastroenterol. Moti..

[34] Jarrett M.E., Burr R.L., Cain K.C., Hertig V., Weisman P., Heitkemper M.M. (2003). Anxiety and depression are related to autonomic nervous system function in women with irritable bowel syndrome. Dig. Dis. Sci..

[35] Heitkemper M., Jarrett M., Cain K.C., Burr R., Levy R.L., Feld A., Hertig V. (2001). Autonomic nervous system function in women with irritable bowel syndrome. Dig Dis Sci..

[36] Zeytinoglu S., Calkins S.D., Leerkes E.M. (2020). Autonomic nervous system functioning in early childhood: Responses to cognitive and negatively valenced emotional challenges. Dev. Psychobiol..

[37] Shinba T. (2017). Major depressive disorder and generalized anxiety disorder show different autonomic dysregulations revealed by heart-rate variability analysis in first-onset drug-naive patients without comorbidity. Psychiatry Clin. Neurosci..

[38] Wojczynski M.K., North K.E., Pedersen N.L., Sullivan P.F. (2007). Irritable bowel syndrome: A co-twin control analysis. Am. J. Gastroenterol..

[39] Camilleri M., Atanasova E., Carlson P.J., Ahmad U., Kim H.J., Viramontes B.E., Mckinzie S., Urrutia R. (2002). Serotonin-transporter polymorphism pharmacogenetics in diarrhea-predominant irritable bowel syndrome. Gastroenterology.

[40] Pata C., Erdal M.E., Derici E., Yazar A., Kanik A., Ulu O. (2002). Serotonin transporter gene polymorphism in irritable bowel syndrome. Am. J. Gastroenterol..

[41] Jun S.E., Kohen R., Cain K.C., Jarrett M.E., Heitkemper M.M. (2014). TPH gene polymorphisms are associated with disease perception and quality of life in women with irritable bowel syndrome. Biol. Res. Nurs..

[42] Sheehan K., Lowe N., Kirley A., Mullins C., Fitzgerald M., Gill M., Hawi Z. (2005). Tryptophan hydroxylase 2 (TPH2) gene variants associated with ADHD. Mol. Psychiatry.

[43] Ottenhof K.W., Sild M., Levesque M.L., Ruhe H.G., Booij L. (2018). TPH2 polymorphisms across the spectrum of psychiatric morbidity: A systematic review and meta-analysis. Neurosci. Biobehav. Rev..

[44] Wolf M., Musch J., Enczmann J., Fischer J. (2012). Estimating the prevalence of nonpaternity in Germany. Hum. Nat..

